# Etymologia: *Fonsecaea pedrosoi*

**DOI:** 10.3201/eid2907.230114

**Published:** 2023-07

**Authors:** Dayane Moraes, Alexandre Melo Bailão, Mirelle Garcia Silva-Bailão

**Affiliations:** Universidade Federal de Goiás, Goiânia, Goiás, Brazil

**Keywords:** Fonsecaea pedrosoi, etymologia, fungal infections, fungi, chromoblastomycosis, dermatomycoses

***Fonse*** ***caea pedrosoi* [fon-se-se′ə pedro´soi]**

Chromoblastomycosis (CBM) is a neglected tropical disease caused by dematiaceous fungi, mainly *Fonsecaea pedrosoi* (Figure). This disease resembles blastomycosis in the Americas; the prefix indicates causative microorganisms’ pigmentation. In 1911, Pedroso observed histologic pathognomonic findings for CBM. He also observed rounded and brownish elements, now called muriform cells, in skin biopsy specimens from a patient in Goiás, Brazil. In 1915, Medlar described a similar cutaneous infection on the basis of culture and histologic analyses. In 1922, Brumpt reported Pedroso’s findings as *Hormodendrum pedrosoi*. Genus description referred to dendroidal conidial chains.

**Figure:**
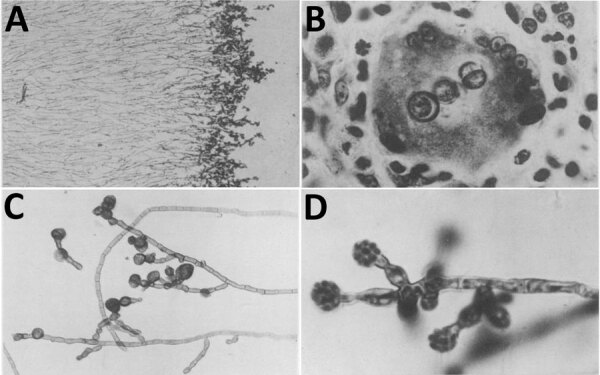
Micrographs showing tissue and culture analyses of a fungal infection case described by Medlar in 1915. A) Section of 5-week-old colony. B) Muriform bodies inside a giant cell. C) Hyphae in a 4-week-old colony. D) Aerial hypha showing numerous, typical sporogenous cells.

Subsequent publications classified this fungus into different genera (*Botrytoides*, *Phialophora*, *Phidoconidiophora*, and *Trichosporium*) on the basis of type of sporulation. Multiple forms of conidiation in dematiaceous fungi contributed to frequent genus changes. Terra et al. described this fungus as *Acrotheca pedrosoi*. In 1936, Negroni renamed this microorganism *Fonsecaea pedrosoi* because *Hormodendrum* and *Acrotheca* reproductive structures were recognized in samples. The genus was named in honor of Fonseca Filho, a Brazilian investigator who made notable contributions to dermatomycoses.
